# Algorithmic management and gig workers' workplace wellbeing: the moderating role of psychological resilience

**DOI:** 10.3389/fpsyg.2026.1960382

**Published:** 2026-10-15

**Authors:** Zha Xiaofang, Zhang Yue, Zhang Dandan, Feng Xinying, Suziana Mat Yasin

**Affiliations:** 1 School of Business and Management, Jiaxing Nanhu University, Jiaxing, China; 2 School of Social Sciences, Universiti Sains Malaysia, Pulau Pinang, George Town, Malaysia

**Keywords:** algorithmic management, challenge appraisal, gig workers, threat appraisal, workplace wellbeing

## Abstract

This study examines how algorithmic management in the gig economy is related to gig workers' workplace wellbeing. Specifically, we explore how algorithmic management corresponds with (positive) challenge appraisal and (negative) threat appraisal. We also examine the moderating role of psychological resilience in these relationships, as well as the implications of appraisal processes for workplace wellbeing. Data were collected from 389 food delivery workers in China. We find that algorithmic management is positively associated with both challenge appraisal and threat appraisal. Challenge appraisal is positively associated with workplace wellbeing, whereas threat appraisal is negatively associated with workplace wellbeing. Psychological resilience strengthens the challenge-appraisal pathway and the corresponding positive indirect relationship with workplace wellbeing, but it does not significantly buffer the threat-appraisal pathway. These findings show that algorithmic management is associated with both positive and negative appraisal processes and that psychological resilience may play different roles across these pathways. The study therefore offers a more nuanced explanation of the relationship between algorithmic management and gig workers' workplace wellbeing.

## 1 Introduction

The rapid growth of the gig economy has changed organizational forms and employment patterns, spurring the emergence of numerous online gig platforms (Jabagi et al., 2025; Wood et al., 2019). Unlike traditional human-based managemential practices, these platforms use algorithmic management to monitor and optimize workers' labor processes (Keegan and Meijerink, 2025; Cameron, 2024; Duggan et al., 2020). Algorithmic management refers to organizational practices that use big data and algorithms to manage workers, partially or fully automating managerial tasks, such as work allocation, performance evaluation, pay determination, and other related tasks (Anicich, 2022; Cameron, 2024; Duggan et al., 2020). The implementation of algorithmic management on online gig platforms has improved efficiency among gig workers, while also introducing increased job demands and potential threats (Zhou et al., 2025; Li et al., 2025; Cram et al., 2022). Consequently, algorithmic management has become a critical factor affecting gig workers' workplace wellbeing.

Today, a growing body of research focuses on the relationship between gig workers' workplace wellbeing and algorithmic management (Deng et al., 2025; Zhang et al., 2022; Lee et al., 2021; Cram et al., 2022; Kinowska and Sienkiewicz, 2023). Some studies argued that algorithmic management can improve work efficiency (Meijerink and Bondarouk, 2023; Panteli et al., 2020), enhance worker' autonomy (Liu and Yin, 2024; Duggan et al., 2022), enhance job resources (Keegan and Meijerink, 2023), raise income (Lee et al., 2021) and promote positive emotional experiences (Deng et al., 2025; Jiang et al., 2025), thereby improving gig workers' workplace wellbeing. Others, however, argued that algorithmic management has a negative impact on gig workers' workplace wellbeing, including labor control (Huang, 2023; Cram et al., 2022), reduced wages (Jiang et al., 2025), social isolation (Alačovska et al., 2025; Wood et al., 2019), overwork (Jabagi et al., 2025; Felix et al., 2023), insufficient sleep (Li et al., 2025), job insecurity (Alačovska et al., 2025), emotional exhaustion (Zayid et al., 2024; Rahman, 2021), and family anxiety (Parent-Rocheleau and Parker, 2022). These inconsistent findings not only obscures the relationship between algorithmic management and gig workers' workplace wellbeing, but also hinder the development of effective practical guidance for enhancing gig workers' workplace wellbeing. Thus, it is important to gain a deeper understanding of the mechanisms underlying the relationship between algorithmic management and gig workers' workplace wellbeing.

The use of algorithms on online gig platforms not only increases job demands for gig workers but also introduces instability and uncertainty into their work arrangements (Shi et al., 2025; Au-Yeung et al., 2025). Some studies thus conceptualize algorithmic management as a stressor that affects gig workers (Krzywdzinski et al., 2024; Zhang et al., 2022; Cram et al., 2022). However, individuals may differ in how they appraise algorithmic management, and these different appraisals may be associated with different levels of workplace wellbeing. This perspective aligns with the core assumptions of stress appraisal theory. Stress appraisal theory emphasizes the importance of individuals' cognitive appraisals in understanding their responses to stress (Folkman et al., 1986). Cognitive appraisal is the process by which people assess the extent to which their environment is relevant to their wellbeing (Folkman et al., 1986; Lazarus, 1991). When people appraise an external environment as harmful to their wellbeing and believe that they lack sufficient resources to cope with it, they are likely to experience negative psychological states and behaviors. In contrast, when people believe that they can cope with this situation effectively, they are more likely to experience positive psychological states and behaviors. Stress appraisal theory suggests that cognitive appraisal is an important link between stressors and individual outcomes (Lazarus, 1991). Hence, stress appraisal theory offers an important theoretical foundation for examining how algorithmic management influences gig workers' workplace wellbeing.

Stress appraisal theory suggests that when people face stressors, they engage in two distinct types of appraisal processes: (positive) challenge appraisals and (negative) threat appraisals (Webster et al., 2011; Mitchell et al., 2019; Peeters et al., 2020). These two appraisals are associated with different psychological and behavioral responses. Focusing on the present study, algorithmic management, on the one hand, increases job demands while also providing opportunities to optimize gig workers' work processes and enhance their efficiency and skills (Chen et al., 2025; Cutolo and Kenney, 2021). In this way, algorithmic management may be associated with stronger (positive) challenge appraisal, thereby enhancing their workplace wellbeing. On the other hand, under strong algorithmic control, gig workers are exposed to multiple sources of stress, such as time pressure, work overload, customer-related demands, compliance monitoring, and feelings of alienation (Zhao et al., 2025; Kinowska and Sienkiewicz, 2023; Liang et al., 2025). These stressors may contribute to stronger (negative) threat appraisal, thereby reducing workers' opportunities to experience positive emotions and undermining their workplace wellbeing. Therefore, algorithmic management may exert dual effects on gig workers' workplace wellbeing through (positive) challenge appraisal and (negative) threat appraisal.

In addition, stress appraisal theory suggests that individuals' cognitive appraisals of stressors are influenced by individual differences, leading to varied coping outcomes (Beeler et al., 2020). Past studies have shown that individual factors, such as psychological resilience, can influence how gig workers perceive algorithmic management, thereby affecting their work behaviors (Chen et al., 2025; Xiongtao et al., 2021). Psychological resilience has been widely recognized as a key psychological resource that helps people to cope with adversity, overcome challenges, and adapt to difficult situations (Fletcher and Sarkar, 2013; Troy et al., 2023). From a trait-based perspective of psychological resilience, individuals with high resilience may not necessarily experience less stress. Instead, they may be more likely to appraise stressors as manageable challenges rather than difficult threats (Troy et al., 2023; Gill and Orgad, 2018). For example, when facing the same algorithmic management pressures, food delivery workers with different levels of psychological resilience may show different cognitive and behavioral patterns. Highly resilient food delivery workers usually have better emotion regulation, stronger confidence in problem-solving, and a greater sense of internal control. Thus, high demands driven by algorithms are more likely to be appraised as challenging opportunities for skill improvement and value demonstration (Cram et al., 2020; Vella and Pai, 2019). Even when confronted with algorithmic uncertainty or biased decisions, they tend to see such experiences as temporary difficulties and actively seek solutions (e.g., optimizing delivery routes), which helps them maintain or even improve their workplace wellbeing (Liang et al., 2025). In contrast, food delivery workers with low psychological resilience possess fewer psychological resources to cope with difficulties and are more likely to feel helpless and lose their sense of control (Vella and Pai, 2019; Troy et al., 2023). Therefore, they are more likely to view algorithmic management as a “threat” to their autonomy and job security. Such threat appraisals increase job insecurity and emotional exhaustion, which may subsequently undermine workplace wellbeing. In sum, this study argues that, when faced with the same algorithmic management stressors, individuals with different levels of psychological resilience are likely to form distinct cognitive appraisals, which may in turn be associated with different levels of workplace wellbeing.

In summary, we propose three research questions. (1) How does algorithmic management associate with gig workers' workplace wellbeing through (positive) challenge appraisal and (negative) threat appraisal? (2) How does psychological resilience moderate the relationships between algorithmic management and (positive) challenge appraisal, and the relationship between algorithmic management and (negative) threat appraisal? (3) How does psychological resilience moderate challenge appraisal and threat appraisal in the relationship between algorithmic management and gig workers' workplace wellbeing?

This study offers an important exploration of the relationship between algorithmic management and workplace wellbeing in the gig economy. First, our study bridges the literature on algorithmic management and stress appraisal theory. We conceptualize algorithmic management as a stressor for gig workers, and identify challenge appraisal and threat appraisal as two cognitive pathways linking algorithmic management with workplace wellbeing. Second, our study examines the moderating role of psychological resilience, with a focus on how it shapes the relationship between algorithmic management and stress appraisals. Third, we further examine whether psychological resilience moderates the indirect relationships between algorithmic management and workplace wellbeing through challenge appraisal and threat appraisal. These findings extend ongoing discussions on algorithmic management and worker wellbeing by highlighting both dual appraisal pathways and the path-specific effects of psychological resilience (e.g., Cram et al., 2022; Deng et al., 2025; Kadolkar et al., 2025). In addition, from a practical perspective, this study provides useful guidance for organizations responsible for the design and implementation of algorithmic management by clarifying how algorithmic management practices affect worker wellbeing.

## 2 Theoretical foundations and research hypotheses

### 2.1 Algorithmic management

With the rapid development of the gig economy and artificial intelligence, algorithmic management has been widely used in organizations, especially on online gig platforms (e.g., Uber in the US, Deliveroo in the UK, and Meituan in China). Algorithmic management refers to a management system in which organizations use big data or digital technologies to assign tasks, evaluate performance, monitor work, and arrange gig workers' schedules (Duggan et al., 2020; Cram et al., 2022; Parent-Rocheleau et al., 2024; Wood et al., 2019). However, research on algorithmic management has lagged behind its rapid adoption in practice. In addition, platform firms often protect their algorithmic technologies through patents, making it difficult to directly observe and measure algorithmic management and evaluate its implications. Existing studies have not reached a consensus on the measurement dimensions of algorithmic management. According to Kellogg et al. (2020), algorithmic management comprises three dimensions: algorithmic direction (e.g., restrictions and recommendations), algorithmic evaluation (e.g., recording and rating), and algorithmic discipline (e.g., rewards and replacement). Möhlmann et al. (2021) conceptualized algorithmic management as comprising two dimensions: algorithmic matching (e.g., supply-demand matching, task allocation, and dynamic pricing) and algorithmic control (e.g., algorithms perform managerial functions, monitoring activities, and nudging). Cram et al. (2022) proposed that algorithmic management consists of gate-keeping control (screening workers' eligibility) and guiding control (e.g., monitoring and performance evaluation). However, these studies are grounded primarily in Western contexts, and tend to conceptualize and measure algorithmic management from a control perspective.

In the context of China's gig economy, recent evidence shows that online gig platforms increasingly adopt hybrid management models that integrate algorithmic management with human assistance (Jiang et al., 2025; Huang, 2023, 2022; Lei, 2021). For example, Huang (2023) found that the two largest food delivery platforms in China-Meituan and Ele.me-have developed a distinctive form of algorithmic management by introducing third-party companies. Specifically, platforms establish cooperation agreements with third-party companies, which provide labor services on their behalf. These third-party firms are granted access to the platforms' back-end management systems, enabling them to organize, guide, and monitor food delivery workers' work processes. In this way, platforms combine algorithm-based coordination with human-assisted management practices, which may facilitate operational coordination. Jiang et al. (2025) took into account the unique characteristics of the Chinese context and, based on in-depth interviews with platform workers and systematic coding of qualitative data, developed a measurement model of algorithmic management comprising four dimensions: assessment and feedback, training and guidance, allocation and pricing, and tracking and monitoring. Given that the focus of our study is food delivery workers in China, we use this model to measure algorithmic management in the present study.

### 2.2 Workplace wellbeing

Improving workplace wellbeing has important implications for both individuals and organizations (Deng et al., 2025). Relevant studies have confirmed that higher levels of workplace wellbeing can enhance employees' work motivation and engagement (Garg and Singh, 2020), which subsequently contribute to improved organizational performance (Markos and Sridevi, 2010; Schneider et al., 2018). Workplace wellbeing refers to employees' overall evaluation of the quality of their job and work experiences. Zheng et al. (2015) divided workplace wellbeing into three dimensions: life wellbeing, work wellbeing, and psychological wellbeing, which together provide a comprehensive understanding of individuals' overall wellbeing. They argued that workplace wellbeing not only involves employees' cognitive evaluations and feelings regarding job and life satisfaction, but also encompasses the psychological experiences and levels of satisfaction derived from their work and personal lives.

In traditional workplaces, managers play an important role in shaping employees' workplace wellbeing. Past research has suggested that managers can contribute to employees' workplace wellbeing by providing sufficient resources (Salanova et al., 2005; Shin et al., 2012), granting work autonomy and control (Harley, 1999), ensuring balanced workloads (Klasmeier and Lehmann-Willenbrock, 2024), and offering adequate job security and working conditions (Guest, 2017; Wilson et al., 2004). With the increasing use of algorithmic management in organizations, some organizations have begun to incorporate algorithmic management into traditional management practices (Goods et al., 2019). However, the implications of algorithmic management for gig workers' workplace wellbeing remain limited, and existing studies have yet to uncover the underlying mechanisms linking the two. Accordingly, this study focuses on exploring how algorithmic management is associated with gig workers' workplace wellbeing.

### 2.3 Algorithmic management and workplace wellbeing

Algorithmic management is closely related to gig workers' work environments, bringing both development opportunities and higher work challenges (Connelly et al., 2021; Deng et al., 2025; Keegan and Meijerink, 2023). On the one hand, algorithmic management assigns tasks and optimizes work processes through big data, which allows gig workers to focus on higher-value aspects of their work (Schor et al., 2024; Duggan et al., 2020). For example, food delivery workers can focus on optimizing delivery routes and strategies, which improves their work efficiency. This change not only enhances efficiency but also provides them with greater motivation and autonomy, further improving their workplace wellbeing under algorithmic management (Newlands, 2021; Rahman, 2021). On the other hand, online gig platforms impose tight algorithmic control over workers through continuous data collection and behavioral monitoring (Parent-Rocheleau and Parker, 2022; Huang, 2023). To meet algorithm-set performance targets, gig workers have to continuously adjust their work behaviors to comply with platform requirements. This process not only accelerates the work pace and increases work intensity, but also undermines the humanized nature of work, leading to feelings of work alienation and reducing workplace wellbeing (Bucher et al., 2021). Therefore, algorithmic management can both create opportunities that enhance gig workers' workplace wellbeing and generate threats and pressures that undermine it.

#### 2.3.1 The positive effects of algorithmic management: activating positive appraisal pathways to workplace wellbeing

Stress appraisal theory suggests that when individuals face stressors, they form different appraisals based on their subjective experiences and then adopt different coping strategies (Peeters et al., 2020). These appraisals take two forms: (positive) challenge appraisal and (negative) threat appraisal. In an other word, individuals' responses to stressors are not determined solely by the objective characteristics of stressors, but also by how they evaluate the potential impacts of stressors on their goals, available resources, and coping abilities (Lazarus and Folkman, 1984; Kilby et al., 2018). Therefore, the same stressor may lead to different appraisal outcomes because individuals may interpret and evaluate it differently (Skinner and Brewer, 2002; Tuckey et al., 2015). In the context of algorithmic management, the same algorithmic practices may be perceived as either supportive resources or demanding constraints (Xiaofang et al., 2026). When gig workers focus on the resources and opportunities provided by algorithms, they may be more likely to develop challenge appraisal. However, when they focus on monitoring, performance pressure, and reduced autonomy, they may be more likely to develop threat appraisal.

Challenge appraisal refers to a situation in which workers view stressors as challenges, and focus on the opportunities and personal growth associated with their work (Kim and Beehr, 2020; Mitchell et al., 2019). Stress appraisal theory suggests that individuals are more likely to form a (positive) challenge appraisal when they view changes at work as beneficial in terms of rewards, personal development, and their goals and wellbeing (Rice and Busby, 2023; Liu et al., 2021; Majeed and Qazi, 2021). Algorithmic management adopted by online gig platforms provides gig workers with new resources and data support for decision-making, which helps gig workers better match tasks with their work needs (Liu and Yin, 2024; Gal et al., 2020; Rahman, 2021). As a result, gig workers tend to hold positive views of algorithmic management, perceiving it as a useful assistant in their work. By relying on algorithms, they can complete work tasks more efficiently (Zhang et al., 2025), which makes them more likely to appraise algorithmic management as a challenge rather than a threat. Furthermore, online gig platforms use algorithmic management to provide real-time evaluation and feedback on gig workers' work behaviors. For example, they offer rewards and incentives, adjust compensation based on performance, and assign service-level rankings (Cini, 2023). These mechanisms turn abstract work performance into clear and comparable metrics, which can partly make high-intensity work feel like a game (Behl et al., 2022; Pereira et al., 2024; Behl et al., 2021). Hence, algorithmic management can not only improve gig workers' work efficiency but also enhance the gamified experience of their work, which may encourage them to form a challenge appraisal. Accordingly, we propose the following hypothesis:

H1: Algorithmic management is positively associated with challenge appraisal.

When individuals appraise work-related stressors as challenges, they are more likely to experience positive, optimistic, and enthusiastic psychological states (Beeler et al., 2020; Rice and Day, 2022; Zhang and Parker, 2022). Challenge appraisal also strengthens individuals' expectations of goal attainment and high achievement. In such situations, individuals focus on the opportunities and personal growth they can gain from their work, which is associated with positive emotions, attitudes, and behaviors (Scheuer et al., 2016). Hence, when gig workers view algorithmic management as a challenge, they are more likely to regard it as beneficial to their work and focus on the opportunities embedded in stressful work situations. This, in turn, fosters positive work experiences and enhances workplace wellbeing (Bliese et al., 2017). Specifically, when gig workers perceive algorithmic management as a challenge, they are more likely to focus on self-improvement, which motivates them to engage in behaviors aimed at growth and development. For example, when food delivery workers perceive algorithmic management as a challenge, they may plan more efficient delivery routes to improve work efficiency (Pieczka and Miszczynski, 2025). When they are able to apply and develop their abilities at work, they may experience higher workplace wellbeing. In addition, challenge appraisal promotes goal attainment and strengthens expectations of high achievement. Gig workers who engage in challenge appraisal tend to set higher goals and hold stronger expectations regarding the potential benefits of algorithmic management. Such positive expectations help satisfy their need for achievement and foster positive emotions toward realizing their potential, which may further contribute to workplace wellbeing. Therefore, we further propose that challenge appraisal mediates the positive relationship between algorithmic management and gig workers' workplace wellbeing. Accordingly, we propose the following hypotheses:

H2: Challenge appraisal is positively associated with gig workers' workplace wellbeing.
H3: Algorithmic management is positively associated with gig workers' workplace wellbeing through challenge appraisal.

#### 2.3.2 The negative effects of algorithmic management: threatening negative appraisal pathways to workplace wellbeing

Threat appraisal refers to a situation in which employees view external stressors as threats, feel worried about the future, and focus on the difficulties and potential losses they may face in their work context (Lazarus and Folkman, 1984; Mitchell et al., 2019). As mentioned above, individuals may form different cognitive appraisals in response to the same stressors. Although algorithmic management can provide gig workers with work resources, it also contains controlling features that may increase perceived work demands and constraints (Cram et al., 2022; Zhao and Liu, 2024). When gig workers focus on these controlling aspects of algorithmic management, they may perceive algorithms as a source of pressure that threatens their work experience and personal development. Accordingly, in addition to challenge appraisal, gig workers may also interpret algorithmic management as a threat. According to stress appraisal theory, when individuals perceive external stressors as hindering the achievement of work goals and as unfavorable to personal growth, they are more likely to engage in threat appraisal (Kraimer et al., 2022; Liu et al., 2022). On online gig platforms, algorithmic management is used to track and monitor gig workers' work processes. For example, food delivery platforms track workers' geographic locations and task progress, observe service behavior, and record work-related behavioral patterns (Wood and Lehdonvirta, 2023; Vallas and Schor, 2020). Such algorithmic management intensifies perceptions of algorithmic control, high work pressure, and limited emotional interaction among gig workers (Gal et al., 2020). Therefore, gig workers may come to view algorithmic management as a constraint on their work development, which triggers threat appraisal. Based on the above reasoning, we propose the following hypothesis:

H4: Algorithmic management is positively associated with threat appraisal.

Stress appraisal theory suggests that individuals who make threat appraisal tend to focus on possible losses and harms from stressors, which makes them more likely to develop negative and pessimistic attitudes and behaviors (Spurk et al., 2021; Gerich and Weber, 2020). When individuals believe that they do not have enough resources to meet demands, their capacity to cope with stress becomes weakened. As a result, they may have poorer coping outcomes, such as negative emotions and work withdrawal (Gerich and Weber, 2020). These negative coping outcomes further erode individuals' positive work experiences and undermine their workplace wellbeing. Thus, threat appraisal is expected to be negatively associated with gig workers' wellbeing at work.

More specifically, first, the rules and procedures established by online gig platforms constrain how work tasks should be completed and the standards that must be met, thereby generating work-related pressure (Nilsson et al., 2025). For example, strict time requirements may force food delivery workers to take risky behaviors, such as running red lights, which can increase dissatisfaction with algorithmic management and then undermine workplace wellbeing (Bartel et al., 2019). Second, the electronic monitoring systems underlying algorithmic management (e.g., real-time location tracking and service monitoring) continuously monitor workers' labor processes and are likely to trigger threat appraisal (Wiener et al., 2023; Huang, 2022). For instance, continuous electronic tracking of service attitudes and task progress may undermine gig workers' sense of autonomy, causing them to feel controlled by the system rather than making their own decisions at work (Wood et al., 2019). This subsequently reduces their workplace wellbeing. Third, punitive measures in algorithmic management also impose substantial pressure on gig workers. For example, food delivery workers may be subject to fines or even account suspension following customer complaints, negative comments, or minor rule violations (Chen et al., 2025). Such practices can cause anxiety and resistance, which may undermine workplace wellbeing. Therefore, higher levels of perceived algorithmic management are more likely to induce threat appraisal of algorithm-related stressors among food delivery workers. This threat appraisal is associated with negative emotions and reduced perceived work autonomy, which may contribute to lower workplace wellbeing. Accordingly, we propose the following hypotheses:

H5: Threat appraisal is negatively associated with gig workers' workplace wellbeing.
H6: Algorithmic management is negatively indirectly associated with gig workers' workplace wellbeing through threat appraisal.

#### 2.3.3 The moderating role of psychological resilience

Drawing on stress appraisal theory, individuals' cognitive appraisal depends on how they understand and interpret stressors in organizational contexts (Lazarus and Folkman, 1984; Mitchell et al., 2019). Differences in psychological resilience may shape individuals' stress appraisals (Mitchell et al., 2019). Prior research has argued that psychological resilience is a key dimension of psychological capital and serves as an important protective factor when individuals face adversity (Vella and Pai, 2019). Psychological resilience significantly influences how individuals perceive and process information, leading them to form different cognitive and emotional responses when facing the same situation or event (Peker and Cengiz, 2022; Cooper et al., 2014). Accordingly, when confronted with algorithmic management practices on online labor platforms, individuals with different levels of psychological resilience are likely to form different cognitive appraisals. Thus, we examine the moderating role of psychological resilience.

Individuals with higher psychological resilience are better able to strengthen the positive relationship between algorithmic management and challenge appraisal (Kuhlman et al., 2021; Vella and Pai, 2019). With higher psychological resilience, workers are more able to reinterpret the pressures associated with algorithmic management. For example, when food delivery workers with high psychological resilience face high performance targets and complex tasks set by algorithms, they are more likely to see them as opportunities for growth rather than as threats (Behl et al., 2022; Pereira et al., 2024). Such workers tend to direct their attention toward the informational support, efficiency gains, and potential rewards enabled by algorithms, which facilitates the formation of challenge appraisal. In addition, individuals with high psychological resilience typically possess stronger problem-solving beliefs and emotion regulation capacities (Ahmed et al., 2022; Den Hartigh and Hill, 2022). For instance, food delivery workers with higher resilience are may be more confident in their ability to optimize delivery routes through effort and strategic adjustment to meet algorithmic requirements (Zhang et al., 2025). In contrast, when psychological resilience is low, gig workers are more likely to focus on task difficulty and the anxiety associated with high performance demands. They tend to attend more to the perceived costs and potential losses associated with algorithmic management and to hold more negative expectations about future outcomes. As a result, they are less likely to appraise algorithmic management as a positive a challenge. Hence, we propose the following hypothesis:

H7a: Psychological resilience moderates the relationship between algorithmic management and challenge appraisal.

Individuals with high psychological resilience tend not only to cope more effectively with stress, but also to recognize opportunities for growth in challenging situations (Troy et al., 2023; Den Hartigh and Hill, 2022; Chen et al., 2017). This positive cognitive orientation helps individuals value the sense of competence and control they gain from overcoming work-related challenges (Zhang et al., 2025). For example, food delivery workers may view algorithmic management as a motivating force that supports their skill development and encourages them to focus on opportunities, growth, and potential gains. Even though algorithmic management imposes work demands that exceed their immediate capacity (e.g., an excessive number of delivery orders), these workers may still believe that, with the technical support and resources provided by algorithms, they are capable of addressing these challenges and achieving work goals (Cram et al., 2022). This may encourage individuals to develop challenge appraisal of algorithmic management, thereby enhancing their positive emotions, and improves their workplace wellbeing. In contrast, individuals with lower psychological resilience are more likely to view algorithmic management as a burden beyond their ability to cope. As a result, they are less likely to develop challenge appraisal, which may be associated with fewer positive emotions and lower workplace wellbeing. Accordingly, we propose the following hypothesis:

H7b: Psychological resilience positively moderates the mediating effect of challenge appraisal in the relationship between algorithmic management and workplace wellbeing.

Furthermore, high psychological resilience can buffer the negative relationship between algorithmic management and threat appraisal. Specifically, psychological resilience provides individuals with important cognitive and emotional resources that enable them to resist and reshape threat perceptions caused by algorithmic management (Kuhlman et al., 2021; Chen et al., 2017). According to stress appraisal theory, threat appraisals are influenced by both the characteristics of stressors and individuals' evaluations of their coping resources and capabilities (Lazarus and Folkman, 1984). As an important personal resource, psychological resilience enhances individuals' ability to adapt to stressful situations. It helps individuals perceive greater coping capability, thereby reducing the likelihood of interpreting stressful events as threats.

Specifically, individuals with high psychological resilience possess stronger cognitive restructuring abilities (Feldman, 1981; Kuhlman et al., 2021; Xiongtao et al., 2021). When faced with strict monitoring by algorithms (e.g., real-time location tracking) and potential penalties (e.g., late payment deductions), they are more likely to make benign attributions. For example, food delivery workers may attribute work problems to temporary and external objective factors (e.g., traffic jams) rather than attributing them to their own incompetence (e.g., “I am not capable of doing this job”). Such attribution patterns prevent them from generalizing isolated negative events into negative judgments about their own professional abilities, thus reducing the impact of threat appraisal in response to algorithmic management. Second, individuals with high psychological resilience are better able to manage and conserve their psychological resources (Guo, 2025; Den Hartigh and Hill, 2022. For example, faced with work-related stress and uncertainty arising from algorithmic management, food delivery workers with high psychological resilience are able to more effectively suppress negative emotions such as anxiety and frustration. Therefore, even when they perceive algorithm-related pressure, they are more likely to remain relatively calm and rational, directing their attention toward problem solving rather than excessive worry. This may substantially reduce their tendency to appraise algorithmic pressure as a personal threat.

However, stress appraisal theory also suggests that individuals' evaluations of their coping abilities are influenced by the characteristics of stressful situations (Lazarus and Folkman, 1984). In the context of algorithmic management, some algorithmic control mechanisms may represent relatively stable external work constraints that are difficult for workers to change. For example, real-time monitoring, performance requirements, and reward and penalty systems may directly influence workers' task allocation, income opportunities, and work autonomy (Wood et al., 2019). When workers perceive these algorithmic demands as beyond their control, even those with high psychological resilience may find it difficult to completely reduce the threat perceptions arising from such external constraints. Therefore, although psychological resilience can help workers reinterpret algorithmic management-related stress and reduce their tendency to make threat appraisals, its protective effect may be constrained by the external constraints embedded in algorithmic management.

In contrast, individuals with low psychological resilience lack sufficient cognitive restructuring ability and emotional regulation resources, making them more prone to interpreting pressure induced by algorithmic management as threatening (Kuhlman et al., 2021). In such cases, algorithmic management is more readily perceived as a threat, thereby increasing the likelihood of threat appraisal. Therefore, psychological resilience may shape how workers interpret and respond to algorithmic management-related stress, thereby reducing their tendency to make threat appraisals. Accordingly, we propose the following hypothesis:

H7c: Psychological resilience negatively moderates the relationship between algorithmic management and threat appraisal.

In addition, psychological resilience may weaken the negative indirect effect of algorithmic management on workplace wellbeing through threat appraisal. Specifically, when facing algorithmic management, gig workers with high psychological resilience possess stronger emotional regulation and problem-solving abilities, enabling them to better cope with the intensified work demands (e.g., excessive workload and time pressure) imposed by algorithmic management. Therefore, they tend to interpret algorithmic management situations in a relatively positive and rational way, rather than viewing them as an unbearable threat (Xiongtao et al., 2021). In other words, psychological resilience reduces the likelihood of algorithmic management inducing threat appraisal. Since threat appraisal is usually accompanied by negative emotions such as fear and anxiety, highly resilient individuals experience fewer of these negative emotions, thus reducing the likelihood of declines in workplace wellbeing. In contrast, gig workers with low psychological resilience often lack effective emotional regulation and cognitive restructuring abilities. They are therefore more likely to perceive the job demands and pressures imposed by algorithmic management as overwhelming threats. Consequently, they are more likely to form threat appraisals and exhibit avoidance or resistance tendencies, which may further contribute to lower workplace wellbeing. Accordingly, we propose the following hypothesis:

H7d: Psychological resilience negatively moderates the mediating role of threat appraisal in the relationship between algorithmic management and workplace wellbeing.

To sum up, this study develops a theoretical model of the influence of algorithmic management on gig workers' workplace wellbeing (see Figure 1).

**Figure 1:**
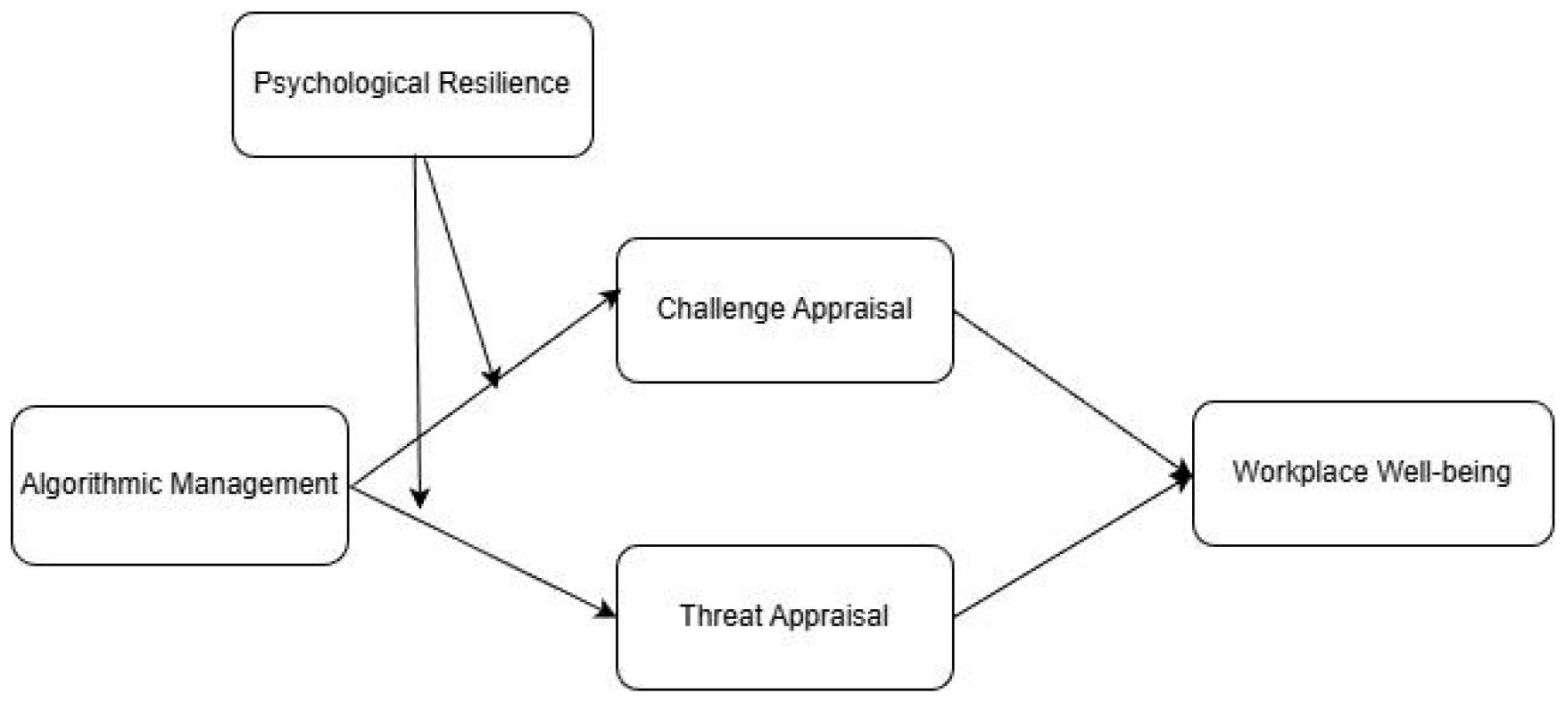
Theoretical model.

## 3 Methodology

### 3.1 Questionnaire design

We used a questionnaire survey to empirically test the proposed hypotheses. Questionnaire surveys are frequently used to obtain data when conducting empirical research. The more rigorous the questionnaire design and measurement process, the stronger the explanatory power of the research results (Saris and Gallhofer, 2014). Thus, a well-designed questionnaire and a scientific survey process contribute to improving the reliability and validity of the study, thereby ensuring credible results and conclusions. Therefore, we carefully designed the research questionnaire.

The questionnaire in this study consists of a main section and a demographic information section. The main section includes the independent variable (algorithmic management), mediating variables (challenge appraisal and threat appraisal), moderating variable (psychological resilience), and dependent variable (workplace wellbeing). The demographic information section included information such as gender, age, marital status, job type, educational level, and monthly income. All constructs were measured using a 7-point Likert scale (1 = Strongly disagree, 7 = Strongly agree), where participants rated each item accordingly. The 7-point Likert scale provides higher resolution and sensitivity than a 5-point scale, and can capture the respondents' psychological states and behavioral tendencies in more detail. Therefore, it improves the accuracy and reliability of the collected data (Joshi et al., 2015). The detailed measurement items, item codes, and sources for all variables in this study are presented in Appendix A.

The questionnaire was originally developed in English and was translated using a translation-back-translation procedure to ensure equivalence in item meanings. Specifically, we invited three professional translators to conduct the translation and back-translation procedures, ensuring accurate semantic representation of the measurement items. In addition, before the formal survey, we conducted a pilot test by distributing 50 questionnaires and received 37 valid responses. Cronbach's α and principal component analysis were used to assess the reliability and validity of the measurement scales. The results showed that the Cronbach's α values of all five scales exceeded 0.7, and all factor loadings were above 0.5, suggesting satisfactory reliability and construct validity. Furthermore, based on feedback from our pilot test, items with unclear wording, ambiguity, or limited comprehensibility were discussed and revised. The final version was then used for the formal survey.

### 3.2 Data collection process

Data were collected from August to November 2025 in Shanghai, Nanjing, and Suzhou. The participants were food delivery workers aged 18 years or older. To recruit participants, we first contacted the regional managers of food delivery platforms in these three cities. With the assistance of these managers, the research team gained access to delivery stations in the three cities and invited food delivery workers to participate in the study. We then shared the recruitment information and online survey link with eligible delivery workers. The managers only assisted with recruitment and did not influence the workers' decisions about whether to participate.

Before the data collection, all participants were informed of the purpose of the study, the content of the survey, and the procedures for participation. The researchers explicitly stated that participation was entirely voluntary and that participants could withdraw from the study at any time. The research team also assured participants that all collected data would be used solely for academic purposes, and that their personal information and responses would be kept strictly confidential and would not be accessed by platform managers or any other third parties.

To reduce the possible impact of common method bias (CMB; Podsakoff et al., 2012), data were collected at two time points. Specifically, at Time 1, a total of 657 participants entered the survey. Participants completed a questionnaire measuring demographic characteristics, the independent variable (algorithmic management), and the moderating variable (psychological resilience). After screening, 86 questionnaires were excluded, leaving 571 valid Time 1 responses, including 206 from Shanghai, 178 from Nanjing, and 187 from Suzhou. To match responses from the same participant across the two waves, participants were asked to provide the last four digits of their mobile phone number as a temporary matching identifier. This identifier was used only to match the two questionnaires and was deleted after matching was completed. The final dataset used for statistical analysis did not contain these four digits or any other information that could directly identify the participants.

Three weeks after the first survey, participants who completed the Time 1 survey were invited to complete a second questionnaire assessing the mediating variables (challenge appraisal and threat appraisal) and the dependent variable (workplace wellbeing). A total of 496 participants completed the Time 2 survey. Some participants did not complete the second survey because they had left their jobs or declined to continue participating. In addition, questionnaires completed in less than 5 minutes were excluded based on the predefined data-quality criterion. This five-minute threshold was established before formal data collection based on the actual questionnaire completion times observed in the pilot study. Finally, 389 valid questionnaires were successfully matched across the two waves, including 153 from Shanghai, 115 from Nanjing, and 121 from Suzhou. The retention rate across the two waves was 68.1%. Upon completion of each survey wave, participants received CNY 20 (approximately USD 3) as compensation.

In this survey, we found that the majority of food delivery workers in China are unmarried, accounting for over 60% of the sample (62.2%). The sample is predominantly male (86.9%), with a large proportion aged between 18 and 25 years (43.2%). More than 80% of respondents work full time (82.0%). In terms of education, most participants have completed high school (40.1%), and monthly income is primarily concentrated in the range of CNY 5,001–6,000 (37.5%; see Table 1). The demographic characteristics of our sample are consistent with those reported in previous studies and reflect the general profile of food delivery workers in China. For example, Lei (2021) reported that Chinese food delivery workers are predominantly male (92%), are mostly in their twenties, and typically have a middle school or high school education. Similarly, Huang (2023) found that the vast majority of Chinese food delivery workers are unmarried men, with 79% engaged in full-time work.

**Table 1:** Sample characteristics (*N* = 389).

Feature	Category	*N*	Proportion (%)	Feature	Category	*N*	Proportion (%)
Gender	Male	338	86.9	Educational level	Primary school	14	3.6
	Female	51	13.1		Junior high school	138	35.5
Age	18–25	168	43.2		High school	156	40.1
	26–35	140	36.0		College	74	19.0
	36–45	40	10.3		Above college	7	1.8
	46–55	28	7.2	Monthly Income	Below 3,000	12	3.1
	above 55	13	3.3		3,000–4,000	37	9.5
Work type	Part-time	70	18.0		4,001–5,000	89	22.9
	Full-time	319	82.0		5,001–6,000	146	37.5
Marital status	Unmarried	242	62.2		Above 6,000	105	27.0
	Married	111	28.5				
	Remarried	10	2.6				
	Divorced	19	4.9				
	Widowed	7	1.8				

**1 USD ≈7 CNY**.

### 3.3 Measurement of variables

(1) Algorithmic management. We adopted the scale developed by Jiang et al. (2025), which consists of 14 items. A sample item is “The algorithm provides me with substantial informational support related to job task completion” (Cronbach's α = 0.894).
(2) Challenge appraisal. Challenge appraisal was measured using the challenge appraisal sub-scale developed by LePine et al. (2016). Because this study focuses on algorithmic management as a specific work stressor, the original items were adapted so that participants evaluated algorithmic management as the focal stressor. The core meaning of the original scale was retained. This scale consisted of three items. A sample item is “Algorithmic management helps me overcome difficulties at work” (Cronbach's α = 0.838).
(3) Threat appraisal. Based on the challenge-threat stress appraisal scale developed by LePine et al. (2016), As with the challenge appraisal scale, the items were adapted so that algorithmic management was the focal work stressor, without changing the core meaning of the original scale. It consists of three items, for example, “Algorithmic management restricts my achievement of personal goals and career development” (Cronbach's α = 0.870).
(4) Workplace WellBeing. Workplace wellbeing was measured using the scale developed by Zheng et al. (2015). Based on the research objectives, the workplace wellbeing subscale was adopted, consisting of six items. Sample items include “I like my current job” and “Overall, I am satisfied with my current work situation” (Cronbach's α = 0.868).
(4) Psychological Resilience. Psychological resilience was measured using the resilience scale developed by Luthans and Youssef-Morgan (2007). The scale comprised six items. A sample item is “When I encounter difficulties at work, I can always find ways to solve them.” Items 2 and 4 were reverse-coded before data analysis to ensure that all items were scored in the same direction (Cronbach's α = 0.887).
(5) Control variables. Consistent with past studies, individual gender, age, educational level, job type (part-time/full-time), marital status, and monthly income were selected as control variables (Cram et al., 2022; Deng et al., 2025). These demographic characteristics may be related to individuals' work experiences and workplace wellbeing and were therefore included as control variables. Specifically, gender was coded as 1 = male and 2 = female, and job type was coded as 1 = part-time and 2 = full-time. Age was coded as 1 = 18–25 years, 2 = 26–35 years, 3 = 36–45 years, 4 = 46–55 years, and 5 = over 55 years. Educational level was coded as 1 = primary school, 2 = junior high school, 3 = high school, 4 = college, and 5 = above college. Monthly income was coded as 1 = below CNY 3,000, 2 = CNY 3,000–4,000, 3 = CNY 4,001–5,000, 4 = CNY 5,001–6,000, and 5 = above CNY 6,000. Due to the marital status is a nominal variable, we used dummy coding with unmarried participants as the reference group. Four dummy variables were created for married, remarried, divorced, and widowedparticipants.

## 4 Results

### 4.1 Reliability and validity analysis

We used SPSS 26.0 and AMOS 24.0 to test the reliability and validity of the measurement instruments. Reliability was evaluated using Cronbach's α. As shown in Table 2, which reports the reliability and validity results for all variables, the overall Cronbach's α values was 0.888, and the Cronbach's α values for all constructs exceeded 0.80, indicating good internal consistency. The composite reliability (CR) values were all above 0.70, and the average variance extracted (AVE) values were all above 0.50. These results indicate good reliability and validity.

**Table 2:** Reliability and validity analysis of variables.

Variable	Item	Standardized estimate	Cronbach's α	KMO	CR	AVE
Algorithmic management	Assessment and feedback	0.777	0.894	0.898	0.842	0.571
	Training and guidance	0.739				
	Allocation and pricing	0.748				
	Tracking and monitoring	0.758				
Assessment and feedback	AF1	0.757	0.827	0.804	0.829	0.548
	AF2	0.706				
	AF3	0.772				
	AF4	0.725				
Training and guidance	TG1	0.772	0.830	0.808	0.832	0.554
	TG2	0.725				
	TG3	0.755				
	TG4	0.723				
Allocation and pricing	AP1	0.814	0.843	0.728	0.844	0.643
	AP2	0.778				
	AP3	0.813				
Tracking and monitoring	TM1	0.772	0.807	0.714	0.808	0.583
	TM2	0.743				
	TM3	0.776				
Challenge appraisal	CA1	0.825	0.838	0.725	0.838	0.634
	CA2	0.773				
	CA3	0.789				
Threat appraisal	TA1	0.837	0.870	0.740	0.870	0.691
	TA2	0.814				
	TA3	0.843				
Workplace wellbeing	WWB1	0.716	0.868	0.876	0.869	0.526
	WWB2	0.736				
	WWB3	0.721				
	WWB4	0.725				
	WWB5	0.734				
	WWB6	0.720				
Psychological resilience	PR1	0.728	0.887	0.869	0.887	0.566
	PR2	0.768				
	PR3	0.748				
	PR4	0.784				
	PR5	0.752				
	PR6	0.733				

Algorithmic management was modeled as a second-order reflective construct with four first-order dimensions: assessment and feedback, training and guidance, allocation and pricing, and tracking and monitoring. The standardized loadings of the four first-order dimensions on the second-order algorithmic management factor were 0.777, 0.739, 0.748, and 0.758, respectively (*p* < 0.001). For the overall algorithmic management construct, Cronbach's α was 0.894, CR was 0.842, and AVE was 0.571. The CR and AVE of the second-order algorithmic management construct were calculated based on the standardized loadings of the four first-order dimensions. Specifically, AVE was calculated from the squared loadings, whereas CR was calculated using the loadings and their corresponding error variances. For the four first-order dimensions, Cronbach's α values ranged from 0.807 to 0.843, CR values ranged from 0.808 to 0.844, and AVE values ranged from 0.548 to 0.643. All values met the recommended criteria. These results indicate good internal consistency, composite reliability, and convergent validity for algorithmic management and its four first-order dimensions. In addition, the KMO values for all constructs were above 0.70, indicating that the data were suitable for further factor analysis.

To further assess the second-order measurement structure of algorithmic management, we compared the second-order factor model with a correlated four-factor model and a one-factor model (see Table 3). The results show that the second-order model had a good fit to the data (χ^2^/*df* = 1.120, GFI = 0.971, NFI = 0.967, IFI = 0.996, TLI = 0.995, CFI = 0.996, RMSEA = 0.018, SRMR = 0.029). The chi-square difference between the second-order model and the correlated four-factor model was not significant (Δχ^2^ = 1.850, Δ*df* = 2, *p* = 0.396). This shows that adding a second-order algorithmic management factor did not significantly reduce model fit. In contrast, the one-factor model showed a much poorer fit. These results, along with the theoretical basis and model parsimony, support modeling algorithmic management as a second-order construct with four first-order dimensions.

**Table 3:** Comparison of measurement models for algorithmic management.

Model	χ^2^/df	GFI	NFI	IFI	TLI	CFI	RMSEA
Second-order model	1.120	0.971	0.967	0.996	0.995	0.996	0.018
Four-factor model	1.125	0.972	0.967	0.996	0.995	0.996	0.018
One-factor model	9.631	0.744	0.698	0.720	0.668	0.719	0.149

In addition, we conducted confirmatory factor analysis (CFA) to examine the measurement model. The results indicate a good model fit (χ^2^/*df* = 1.167, GFI = 0.924, RMSEA = 0.021, CFI = 0.987, NFI = 0.915, IFI = 0.987). The average variance extracted (AVE) values for all constructs exceeded 0.50, suggesting satisfactory construct validity. Furthermore, the composite reliability (CR) values for all constructs were above 0.70, demonstrating good convergent validity. Furthermore, as shown in Table 4, the square roots of the AVE values for each construct were greater than the corresponding inter-construct correlation coefficients, supporting discriminant validity.

**Table 4:** Descriptive statistics and correlation analysis of variables.

Variable	Algorithmic management	Challenge appraisal	Threat appraisal	Workplace wellbeing	Psychological resilience
Algorithmic management	**0.756**				
Challenge appraisal	0.404^***^	**0.796**			
Threat appraisal	0.278^***^	0.069	**0.831**		
Workplace wellbeing	0.238^***^	0.350^***^	−0.140^**^	**0.725**	
Psychological resilience	0.157^**^	0.268^**^	0.137^**^	0.173^**^	**0.752**
Mean	4.792	4.590	4.674	4.857	4.703
Standard deviation	0.922	1.299	1.357	1.154	1.279
Skewness	−0.910	−0.641	−0.488	−0.908	−0.806
Kurtosis	1.050	−0.625	−0.719	−0.091	−0.195
VIF	1.293	1.267	1.100	-	1.095

*p < 0.050.

**p < 0.010.

***p < 0.001.

### 4.2 Common method bias test

To mitigate common method bias (CMB), we adopted a time-lagged design and collected survey data at two different time points. However, as all variables were derived from a single source of self-reported data, CMB may still represent a potential concern. To address this concern, we conducted a series of *post hoc* analyses to test the CMB. First, high correlations among constructs may indicate the presence of CMB (Podsakoff et al., 2012). In our data, the highest correlation among constructs was 0.404 (see Table 4), which was below the threshold of 0.90 (Bagozzi et al., 1991). Second, Harman's single-factor test was used to examine whether a single factor accounted for the majority of the variance. The results showed that the largest factor explained 23.641% of the total variance, which is below 40%, suggesting that CMB was unlikely to be a serious concern. Third, we employed collinearity assessment approach proposed by Kock and Lynn (2012), which has been proven to be an effectiveme thod for detecting CMB (Kock, 2017). All variance inflation factor (VIF) values were below the conservative threshold of 3.3, with the highest VIF being 1.293 (see Table 4). Fourth, following Podsakoff et al. (2003), we used the unmeasured latent method factor technique. We added a common method factor to the measurement model and compared model fit before and after adding this factor. The changes in the fit indices were small: Δχ^2^/*df* = 0.018, ΔGFI = 0.006, ΔCFI = 0.002, ΔTLI = 0.001, ΔIFI = 0.002, ΔNFI = 0.008, and ΔRMSEA = 0.001. All changes were below 0.02, indicating that adding the common method factor did not clearly improve model fit. Fifth, we conducted confirmatory factor analysis (CFA) and compared the hypothesized five-factor model with several alternative models. The five-factor model showed a good fit to the data (χ^2^/*df* = 1.167, GFI = 0.924, NFI = 0.915, IFI = 0.987, TLI = 0.986, CFI = 0.987, RMSEA = 0.021) and fit the data better than the four-factor, three-factor, two-factor, and one-factor models (see Table 5). In particular, the one-factor model showed a much poorer fit, indicating that a single-factor structure could not adequately explain the observed data.

**Table 5:** Comparison of confirmatory factor analysis models.

Model	Factor	χ^2^/*df*	GFI	NFI	IFI	TLI	CFI	RMSEA
Five-factor model	A, B, C, D, E	1.167	0.924	0.915	0.987	0.986	0.987	0.021
Four-factor model	A+B, C, D, E	1.762	0.870	0.871	0.940	0.934	0.939	0.044
Three-factor model	A+B+C, D, E	3.021	0.795	0.777	0.839	0.824	0.838	0.072
Two-factor model	A+B+C+D, E	4.156	0.714	0.692	0.748	0.726	0.746	0.090
One-factor model	A+B+C+D+E	6.222	0.581	0.538	0.582	0.546	0.579	0.116

A, algorithmic management; B, challenge appraisal; C, threat appraisal; D, workplace wellbeing; E, psychological resilience.

In sum, these tests did not show clear evidence of serious common method bias. However, these statistical tests cannot completely rule out the possibility of common method variance. Therefore, the possible influence of common method bias should still be considered when interpreting the results.

### 4.3 Correlation analysis

The descriptive statistics and correlation matrix for all variables are presented in Table 4. The results show that the correlations among algorithmic management, challenge appraisal, threat appraisal, workplace wellbeing, and psychological resilience are generally in the expected directions based on the proposed hypotheses. All correlation coefficients are below 0.70, suggesting that multicollinearity is unlikely to be a serious concern and providing preliminary support for subsequent hypothesis testing.

### 4.4 Structural equation model testing

Structural equation modeling (SEM) was conducted to examine the overall theoretical model and to test the proposed relationships among algorithmic management, challenge appraisal, threat appraisal, workplace wellbeing, and psychological resilience. The model fit indices are reported in Table 6. The results indicate a good model fit (χ^2^/*df* = 1.153, GFI = 0.939, RMSEA = 0.020, NFI = 0.931, CFI = 0.990, TLI = 0.989, IFI = 0.990). In sum, these indices indicate that the proposed theoretical model provides an adequate fit to the data. The standardized path coefficients of the model are presented in Figure 2.

**Table 6:** Fitting result indicators of theoretical model.

Common indicators	χ2	*df*	χ^2^/*df*	GFI	RMSEA	NFI	CFI	TLI	IFI
Criteria for judgment	−	−	< 3	>0.9	< 0.10	>0.9	>0.9	>0.9	>0.9
Value	335.586	291	1.153	0.939	0.020	0.931	0.990	0.989	0.990

**Figure 2:**
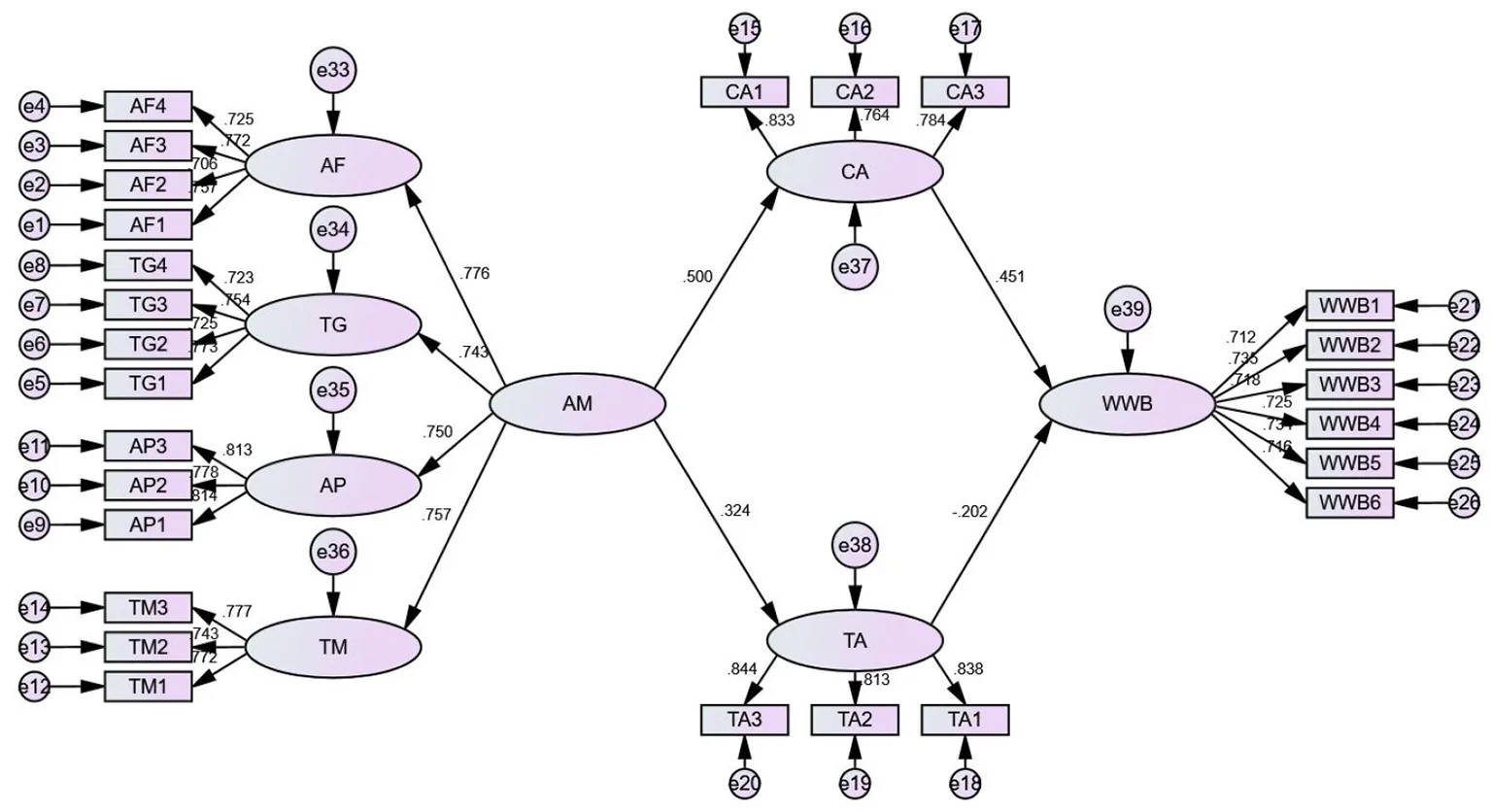
Standardized model diagram.

We further examined the structural paths among the study variables. The results are reported in Table 7. The standard errors (S.E.) ranged from 0.042 to 0.108, and the absolute values of the critical ratios (C.R.) all exceeded 1.96, indicating that the estimated paths were statistically significant. As shown in Table 7, algorithmic management was positively associated with challenge appraisal (β = 0.500, *p* < 0.001), supporting H1. In addition, challenge appraisal was positively associated with workplace wellbeing (β = 0.451, *p* < 0.001), so H2 is supported.

**Table 7:** Hypothesis testing results.

Variable relationship	Path coefficient	S.E.	C.R.	*P*	Test results
Algorithmic management → challenge appraisal	0.500	0.108	7.236	^***^	Supported
Algorithmic management → threat appraisal	0.324	0.103	5.063	^***^	Supported
Challenge appraisal → workplace wellbeing	0.451	0.049	7.230	^***^	Supported
Threat appraisal → workplace wellbeing	−0.202	0.042	−3.604	^***^	Supported

*p < 0.050.

**p < 0.010.

***p < 0.001.

Algorithmic management was also positively associated with threat appraisal (β = 0.324, *p* < 0.001), supporting H4.The path coefficient from algorithmic management to challenge appraisal was larger than that to threat appraisal (β = 0.500 and β = 0.324, respectively), suggesting that algorithmic management exhibited a relatively stronger association with challenge appraisal than with threat appraisal among food delivery workers. Furthermore, threat appraisal was negatively associated with workplace wellbeing (β = −0.202, p < 0.001), supporting H5.

#### 4.4.1 Mediation effect test

Following the approach of Hair et al. (2011), we tested the indirect effects using a bootstrap procedure with 5,000 resamples, and the results are presented in Table 8. The indirect effect of algorithmic management on workplace wellbeing through challenge appraisal was 0.226 (*p* < 0.001), with the 95% confidence interval excluding 0. Thus, H3 was supported. The indirect effect of algorithmic management on workplace wellbeing through threat appraisal was −0.065 (*p* < 0.001), and its 95% confidence interval excluded 0 as well, thereby H6 was supported.

**Table 8:** Results of mediation effect test.

Impact path	Effect	Boot SE	95%Boot		*P*
			LLCI	ULCI	
Algorithmic management → challenge appraisal → workplace wellbeing	0.226	0.044	0.149	0.327	^***^
Algorithmic management → threat appraisal → workplace wellbeing	−0.065	0.022	−0.116	−0.029	^***^

*p < 0.050.

**p < 0.010.

***p < 0.001.

#### 4.4.2 Moderating effect test

We adopted hierarchical regression analysis to test the moderating effects by sequentially entering control variables, the independent variable, the moderating variable, and the interaction term of the independent and moderating variables into the regression models. Prior to analysis, the independent and moderating variables were standardized before constructing the interaction terms to reduce potential multicollinearity.

As shown in Table 9, in Model 3, the interaction between algorithmic management and psychological resilience was positive and statistically significant in challenge appraisal (β = 0.133, *p* < 0.050). The model yielded R = 0.501, *R*^2^ = 0.251, and F = 5.843 (*p* < 0.001), indicating that it explained 25.1% of the variance in challenge appraisal. These results supported H7a. The interaction pattern is illustrated in Figure 3.

**Table 9:** Results of moderation effect test.

Variable	Category	Challenge appraisal	Challenge appraisal	Challenge appraisal	Threat appraisal	Threat appraisal	Threat appraisal
		Model 1	Model 2	Model 3	Model 4	Model 5	Model 6
(Constant)		1.930^**^	1.394^*^	1.378^*^	1.894^**^	1.641^*^	1.637^*^
Gender	Male						
	Female	−0.209	−0.238	−0.262	−0.148	−0.162	−0.168
Age	18–25						
	26–35	0.160	0.108	0.058	−0.149	−0.173	−0.187
	36–45	−0.260	−0.273	−0.304	−0.156	−0.162	−0.170
	46–55	−0.250	−0.219	−0.225	−0.004	0.010	0.009
	Above 55	−0.446	−0.418	−0.426	0.135	0.148	0.146
Job type	Part-time						
	Full-time	0.053	−0.001	−0.041	0.219	0.193	0.182
Marital STATUS	Unmarried						
	Married	−0.007	−0.004	0.019	0.240	0.241	0.247
	Remarried	0.175	0.203	0.240	0.028	0.041	0.051
	Divorced	−0.200	−0.176	−0.145	0.508	0.520	0.528
	Widowed	0.996	0.798	0.747	0.934	0.840	0.827
Education level	Primary school						
	Junior high school	−0.083	−0.213	−0.235	0.317	0.256	0.250
	High school	−0.215	−0.315	−0.343	0.469	0.422	0.414
	College	−0.267	−0.381	−0.419	0.417	0.363	0.353
	Above college	−0.243	−0.370	−0.336	0.697	0.637	0.646
Monthly income	Below 3,000						
	3,000–4,000	0.366	0.338	0.361	0.395	0.382	0.388
	4,001–5,000	0.040	0.063	0.088	0.388	0.399	0.406
	5,001–6,000	0.148	0.113	0.119	0.518	0.501	0.503
	Above 6,000	−0.203	−0.192	−0.164	0.182	0.187	0.195
Algorithmic management		0.576^***^	0.533^***^	0.525^***^	0.378^***^	0.357^***^	0.355^***^
Psychological resilience			0.198^***^	0.217^***^		0.093	0.099
Algorithmic management ^*^ psychological resilience				0.133^*^			0.036
*R*		0.450	0.487	0.501	0.335	0.345	0.347
*R* ^2^		0.202	0.237	0.251	0.112	0.119	0.120
*F*		4.925^***^	5.727^***^	5.843^***^	2.454^***^	2.494^***^	2.389^***^

*p < 0.050.

**p < 0.010.

***p < 0.001.

**Figure 3:**
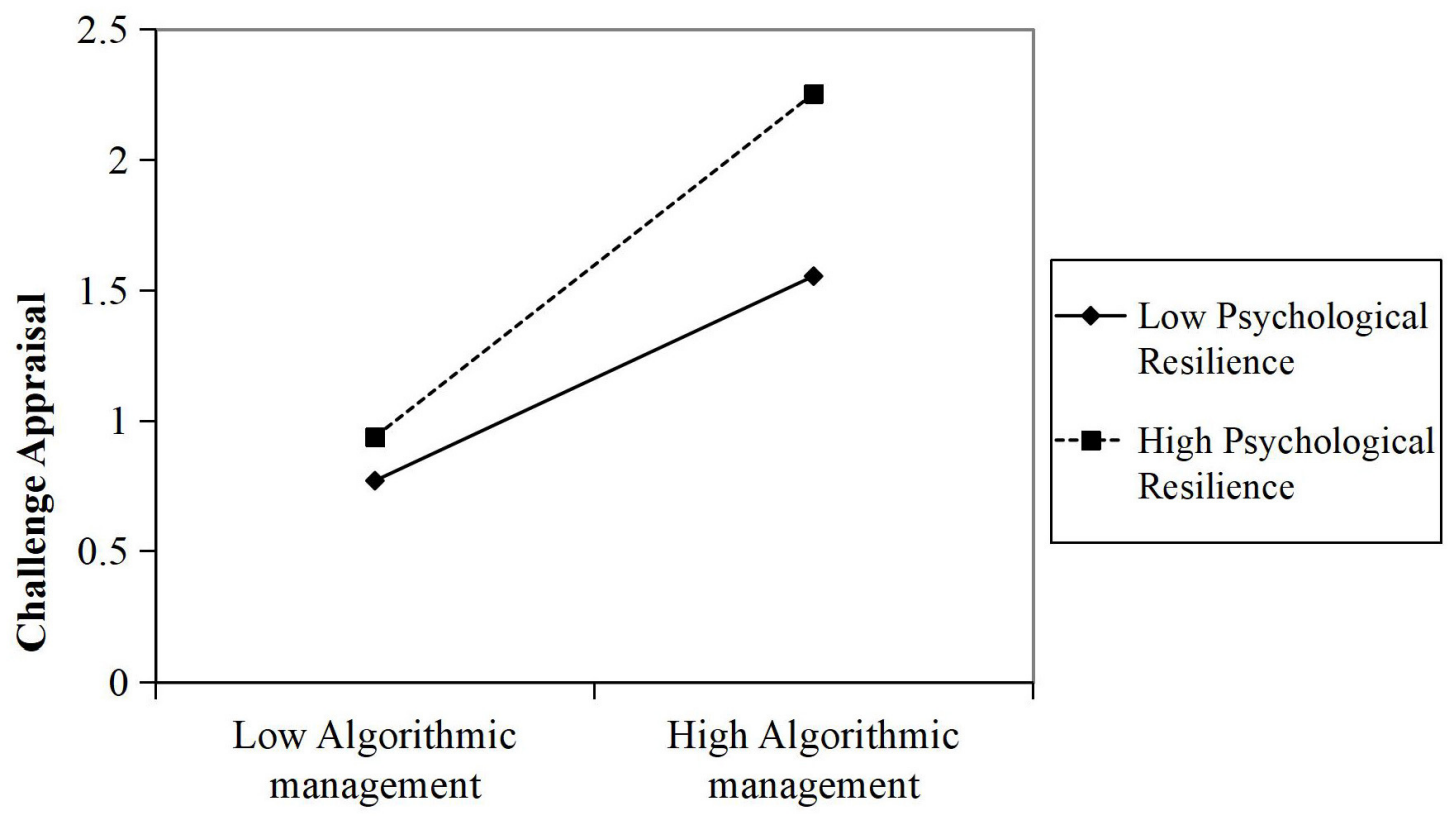
The moderating effect of Psychological Resilience between algorithmic management and challenge appraisal.

In Model 6, the interaction between algorithmic management and psychological resilience was positive but not statistically significant in threat appraisal (β = 0.036, *p* > 0.050). The model yielded *R* = 0.347, *R*^2^ = 0.120, and *F* = 2.389 (*p* < 0.001), indicating that it explained 12% of the variance in threat appraisal. Therefore, H7c is not supported.

#### 4.4.3 Test of moderated mediation effects

We used a moderated mediation model with a bootstrap procedure. And estimated the conditional indirect effects through challenge appraisal and threat appraisal at low, mean, and high levels of psychological resilience. We also calculated the corresponding 95% confidence intervals (CIs). This approach was used to examine the moderating effect of psychological resilience on the mediating roles of challenge appraisal and threat appraisal in the proposed model.

As shown in Table 10, at the low level of psychological resilience (M-1SD), the indirect effect of algorithmic management on workplace wellbeing through challenge appraisal was 0.073 (95% CI = [0.012, 0.118]), which excluded 0. At the medium level, the indirect effect was 0.123 (95% CI = [0.076, 0.171]), also with a confidence interval that excluded 0. At the high level of psychological resilience (M+1SD), the indirect effect was 0.139 (95% CI = [0.085, 0.196]), which excluded 0 as well. These results indicated that the indirect effect of algorithmic management on workplace wellbeing sbecame stronger as psychological resilience increased. Therefore, H7b is supported.

**Table 10:** Moderated mediation effect test.

Mediating pathway	Moderator	Mediating effect	SE.	95% Confidence Interval	
				Lower bound	Upper bound
Algorithmic management → challenge appraisal → workplace wellbeing	M-1SD	0.073	0.027	0.012	0.118
	*M*	0.123	0.025	0.076	0.171
	M+1SD	0.139	0.029	0.085	0.196
Algorithmic management → threat appraisal → workplace wellbeing	M-1SD	−0.052	0.024	−0.104	−0.013
	*M*	−0.061	0.019	−0.101	−0.028
	M+1SD	−0.063	0.022	−0.111	−0.026

Furthermore, the bootstrap analysis examined the conditional indirect effect of algorithmic management on workplace wellbeing through threat appraisal at different levels of psychological resilience. At the low level of psychological resilience (M-1SD), the indirect effect of algorithmic management on workplace wellbeing through threat appraisal was −0.052 (95% CI = [−0.104, −0.013]), excluded 0. At the medium level, the indirect effect was −0.061 (95% CI = [−0.101, −0.028]), and the confidence interval also excluded 0. At the high level of psychological resilience (M+1SD), the indirect effect was −0.063 (95% CI = [−0.111, −0.026]), also excluded 0. All three confidence intervals excluded 0, indicating a significant negative indirect relationship between algorithmic management and workplace wellbeing through threat appraisal at each level of psychological resilience.

To test whether the indirect effects varied across levels of psychological resilience, we calculated the index of moderated mediation and its 95% bootstrap confidence interval using 5,000 bootstrap resamples (Hayes, 2015). The results are presented in Table 11.

**Table 11:** Index of moderated mediation.

Moderator	Mediation pathway	Index	SE	95% Bootstrap CI	
				Lower	Upper
Psychological resilience	Algorithmic management → challenge appraisal → workplace wellbeing	0.032	0.016	0.009	0.069
	Algorithmic management → threat appraisal → workplace wellbeing	−0.006	0.013	−0.032	0.021

For the ‘algorithmic management → challenge appraisal → workplace wellbeing' pathway, the index of moderated mediation was 0.032 (SE = 0.016), with a 95% bootstrap confidence interval of [0.009, 0.069]. The confidence interval did not include 0, indicating the moderated mediation effect was statistically significant. The positive index indicates that the indirect relationship between algorithmic management and workplace wellbeing through challenge appraisal was stronger at higher levels of psychological resilience.

For the ‘algorithmic management → threat appraisal → workplace wellbeing' pathway, the index of moderated mediation was −0.006 (SE = 0.013), with a 95% bootstrap confidence interval of [−0.032, 0.021]. The confidence interval included 0. Thus, the moderated mediation effect was not statistically significant. This result indicates that psychological resilience did not significantly moderate the indirect association between algorithmic management and workplace wellbeing through threat appraisal. Therefore, H7d was not supported.

### 4.5 Post-hoc analysis of algorithmic management dimensions

We further conducted a *post-hoc* analysis by decomposing algorithmic management into four dimensions: assessment and feedback, training and guidance, allocation and pricing, and tracking and monitoring. We then examined their respective effects on challenge appraisal and threat appraisal. The results are presented in Tables 12 and 13.

**Table 12:** Regression analysis of four dimensions of algorithmic management predicting challenge appraisal.

Independent variable	Category	Unstandardized coefficients		Standardized coefficients	*t*	*P*	Collinearity statistics	
		*B*	S.E.	Beta			Tolerance	VIF
(Constant)		1.821	0.587		3.104	0.002		
Gender	Male							
	Female	−0.253	0.189	−0.066	−1.336	0.183	0.868	1.153
Age	18–25							
	26–35	0.159	0.154	0.059	1.029	0.304	0.647	1.546
	36–45	−0.266	0.266	−0.062	−0.998	0.319	0.543	1.841
	46–55	−0.287	0.309	−0.057	−0.929	0.354	0.555	1.803
	Above 55	−0.445	0.417	−0.062	−1.066	0.287	0.631	1.584
Job type	Part-time							
	Full-time	0.036	0.201	0.011	0.179	0.858	0.597	1.674
Marital status	Unmarried							
	Married	0.039	0.178	0.014	0.219	0.827	0.549	1.821
	Remarried	0.186	0.419	0.023	0.443	0.658	0.806	1.240
	Divorced	−0.040	0.343	−0.007	−0.116	0.908	0.651	1.537
	Widowed	1.032	0.536	0.106	1.926	0.055	0.699	1.431
Education level	Primary school							
	Junior high school	−0.023	0.355	−0.009	−0.066	0.948	0.123	8.112
	High school	−0.125	0.361	−0.047	−0.348	0.728	0.113	8.816
	College	−0.113	0.388	−0.034	−0.292	0.770	0.153	6.552
	Above college	−0.070	0.587	−0.007	−0.119	0.905	0.582	1.719
Monthly income	Below 3,000							
	3,000–4,000	0.409	0.407	0.092	1.004	0.316	0.249	4.021
	4,001–5,000	0.084	0.410	0.027	0.205	0.838	0.120	8.345
	5,001–6,000	0.222	0.409	0.083	0.542	0.588	0.090	11.076
	Above 6,000	−0.158	0.428	−0.054	−0.369	0.712	0.098	10.158
Assessment and feedback		0.366	0.066	0.322	5.528	0.000	0.619	1.614
Training and guidance		0.020	0.065	0.018	0.312	0.755	0.613	1.631
Allocation and pricing		0.136	0.060	0.133	2.279	0.023	0.620	1.612
Tracking and monitoring		0.056	0.064	0.051	0.871	0.384	0.627	1.595
*R*		0.478						
*R* ^2^		0.229						
*F*		4.937 (*p* < 0.001)						

Dependent variable: challenge appraisal.

**Table 13:** Regression analysis of four dimensions of algorithmic management predicting threat appraisal.

Independent variable	Category	Unstandardized coefficients		Standardized coefficients	*t*	*P*	Collinearity statistics	
		*B*	S.E.	Beta			Tolerance	VIF
(Constant)		1.933	0.656		2.948	0.003		
Gender	Male							
	Female	−0.163	0.212	−0.041	−0.771	0.441	0.868	1.153
Age	18–25							
	26–35	−0.161	0.172	−0.057	−0.933	0.352	0.647	1.546
	36–45	−0.161	0.297	−0.036	−0.543	0.587	0.543	1.841
	46–55	0.003	0.346	0.001	0.009	0.993	0.555	1.803
	Above 55	0.095	0.466	0.013	0.203	0.839	0.631	1.584
Job type	Part-time							
	Full-time	0.219	0.224	0.062	0.978	0.329	0.597	1.674
Marital status	Unmarried							
	Married	0.255	0.199	0.085	1.283	0.200	0.549	1.821
	Remarried	0.052	0.468	0.006	0.111	0.911	0.806	1.240
	Divorced	0.512	0.383	0.081	1.336	0.182	0.651	1.537
	Widowed	0.882	0.599	0.086	1.472	0.142	0.699	1.431
Education level	Primary school							
	Junior high school	0.241	0.396	0.085	0.609	0.543	0.123	8.112
	High school	0.404	0.403	0.146	1.002	0.317	0.113	8.816
	College	0.361	0.434	0.104	0.831	0.406	0.153	6.552
	Above college	0.667	0.656	0.065	1.016	0.310	0.582	1.719
Monthly income	Below 3,000							
	3,000–4,000	0.434	0.455	0.094	0.953	0.341	0.249	4.021
	4,001–5,000	0.391	0.458	0.121	0.855	0.393	0.120	8.345
	5,001–6000	0.523	0.457	0.187	1.144	0.253	0.090	11.076
	Above 6,000	0.173	0.478	0.057	0.362	0.718	0.098	10.158
Assessment and feedback		0.112	0.074	0.094	1.512	0.131	0.619	1.614
Training and guidance		0.017	0.073	0.014	0.227	0.821	0.613	1.631
Allocation and pricing		0.088	0.067	0.083	1.325	0.186	0.620	1.612
Tracking and monitoring		0.166	0.071	0.144	2.323	0.021	0.627	1.595
*R*		0.343						
*R* ^2^		0.118						
*F*		2.222 (*p* < 0.001)						

Dependent variable: threat appraisal.

As shown in Table 12, different dimensions of algorithmic management exhibited distinct predictive patterns for challenge appraisal. Specifically, assessment and feedback had a significant positive effect on challenge appraisal (β = 0.366, *p* < 0.001), and allocation and pricing also showed a significant positive effect (β = 0.136, *p* < 0.05). In contrast, the effects of training and guidance (β = 0.020, *p* > 0.05) and tracking and monitoring (β = 0.056, *p* > 0.05) on challenge appraisal were not statistically significant. The overall model was significant (*F* = 4.937, *p* < 0.001) and explained 22.9% of the variance in challenge appraisal (*R*^2^ = 0.229).

Furthermore, as shown in Table 13, different dimensions of algorithmic management also demonstrated distinct predictive patterns for threat appraisal. Among these dimensions, tracking and monitoring had a significant positive effect on threat appraisal (β = 0.144, *p* < 0.05), whereas the effects of assessment and feedback (β = 0.094, *p* > 0.05), training and guidance (β = 0.014, *p* > 0.05), and allocation and pricing (β = 0.083, *p* > 0.05) were not statistically significant. The overall model was significant (*F* = 2.222, *p* < 0.001) and explained 11.8% of the variance in threat appraisal (*R*^2^ = 0.118). These findings show that the different functional dimensions of algorithmic management may contribute differently to gig workers' cognitive appraisal processes.

### 4.6 Robustness analysis

To examine the robustness of the main findings, we conducted two supplementary analyses. First, we re-estimated the SEM and mediation models with the control variables included. The results showed that, compared with the original model, the model fit indices changed after including the control variables. Specifically, some incremental fit indices (e.g., GFI, NFI, and TLI) decreased; however, the overall model fit remained within an acceptable range (χ^2^/*df* = 2.084, RMSEA = 0.053, CFI = 0.901). Meanwhile, the directions and statistical significance of the main structural paths and indirect effects remained consistent with the original results, indicating that the inclusion of control variables did not substantially alter the core findings of this study.

Second, we tested an alternative model that included a direct path from algorithmic management to workplace wellbeing. The original theoretical model did not specify this direct path because the study focused primarily on cognitive appraisal mechanisms. This did not imply an a priori assumption that no direct association existed between algorithmic management and workplace wellbeing. Model comparison results showed that the partial mediation model with the direct path fit the data significantly better than the original full mediation model, Δχ^2^(1) = 6.943, *p* < 0.010. Further analysis showed that the direct association between algorithmic management and workplace wellbeing was significant (β = 0.191, *p* < 0.050), and the total effect was also significant (β = 0.277, *p* < 0.001). In addition, the indirect effect through challenge appraisal was positive (β = 0.173), whereas the indirect effect through threat appraisal was negative (β = −0.087). The total indirect effect was β = 0.086. These results indicate that, after the direct path was added, a significant direct association between algorithmic management and workplace wellbeing remained, and the overall model fit improved significantly.

## 5 Discussion

We tested a total of ten hypotheses (H1, H2, H3, H4, H5, H6, H7a, H7b, H7c, and H7d). The findings of this study show that algorithmic management is positively associated with both challenge appraisal and threat appraisal. Challenge appraisal is positively associated with workplace wellbeing, whereas threat appraisal is negatively associated with workplace wellbeing. Furthermore, psychological resilience significantly moderates the relationship between algorithmic management and challenge appraisal, such that this positive relationship is stronger at higher levels of psychological resilience. However, psychological resilience does not moderate the relationship between algorithmic management and threat appraisal (H7c). Moreover, its moderating effect on the indirect relationship between algorithmic management and workplace wellbeing through threat appraisal was not statistically significant (H7d). These findings were inconsistent with our expectations. In addition, the robustness analyses further showed that the main findings remained stable after the control variables were included. Moreover, the direct association between algorithmic management and workplace wellbeing remained significant when this path was added to the model. These findings suggest that challenge appraisal and threat appraisal are important cognitive mechanisms linking algorithmic management to workplace wellbeing, but they do not fully account for this association.

### 5.1 Theoretical contributions

Based on stress appraisal theory, this study adopts a “stressors-cognitive appraisal-stress response” perspective to offer a new theoretical lens for explaining the mechanisms linking algorithmic management and workplace wellbeing. Although prior studies have examined the relationship between algorithmic management and gig workers' workplace wellbeing. However, the findings have been mixed and sometimes inconsistent. To further advance this line of research, we identify two cognitive appraisal pathways linking algorithmic management with gig workers' workplace wellbeing.

First, our findings show that algorithmic management is positively associated with both challenge appraisal and threat appraisal, This finding suggests that gig workers may interpret algorithmic management through dual cognitive pathways, involving both positive and negative psychological responses. This finding is consistent with the conclusions of Cram et al. (2020, 2022). It is important to note that Cram et al. (2022) examined perceived technological stressors among U.S. Uber drivers from the perspectives of algorithmic control (i.e., gate-keeping control and guiding control). Our study, however, focuses on algorithmic management as a more integrated managerial construct, and examines how Chinese food delivery workers cognitively appraise algorithm-induced pressure. Although the two studies differ in research context, cultural and institutional background, target population, and conceptual framing, both find that algorithmic work environments may simultaneously trigger positive and negative psychological experiences. This convergence suggests that similar cognitive appraisal mechanisms may exist across different algorithmic work contexts.

Moreover, our findings are also consistent with recent related studies (e.g., Deng et al., 2025; Kinowska and Sienkiewicz, 2023). These studies suggest that gig workers may simultaneously develop positive and negative appraisals of algorithmic management. Therefore, unlike perspectives that view algorithmic management simply as either a beneficial tool or a control mechanism, this study highlights the importance of how workers interpret and evaluate algorithmic management. It provides a more nuanced theoretical perspective for understanding gig workers' psychological experiences under algorithmic management. Furthermore, to better understand how the dual appraisal pathways of algorithmic management emerge, we further examined the effects of its different functional dimensions. In this regard, the overall analysis and dimensional analysis focus on different levels of effects. The overall analysis aims to capture gig workers' overall appraisal of algorithmic management, rather than comparing the relative effects of different dimensions. Therefore, the relationships between algorithmic management and challenge and threat appraisals reflect gig workers' overall perceptions of algorithmic management, rather than indicating that all dimensions of algorithmic management have the same effects. Based on this, the *post-hoc* dimensional analysis further reveals the functional differences within algorithmic management. Specifically, assessment and feedback and allocation and pricing, as support-oriented functions, were more closely associated with challenge appraisal, whereas tracking and monitoring, as a control-oriented function, was more closely associated with threat appraisal. These findings suggest that the dual appraisal pathways of algorithmic management may reflect the different roles played by its functional dimensions.

Notably, although algorithmic management was positively associated with both challenge appraisal and threat appraisal, its association with challenge appraisal was stronger. This suggests that the dual effects of algorithmic management may not be entirely symmetrical. In the food delivery context, algorithmic systems provide task guidance, real-time information, and performance feedback, which may help gig workers improve efficiency and achieve work goals. These functions may be particularly valuable for younger workers or those with limited occupational experience, as they can reduce work uncertainty and support skill development. Therefore, gig workers may be more likely to appraise algorithmic management as a challenge. However, algorithmic monitoring, performance requirements, and reward and penalty mechanisms may still constrain autonomy and trigger threat appraisals. Overall, the relative strength of these two appraisals may depend on how workers evaluate the resources and pressures associated with algorithmic management.

Second, our findings extend the literature on psychological resilience by showing that its role may differ across cognitive appraisal pathways. Existing studies have generally viewed psychological resilience as an important personal psychological resource that helps individuals cope with stress and adversity (Kuhlman et al., 2021; Troy et al., 2023; Vella and Pai, 2019). However, whether psychological resilience can simultaneously influence different types of cognitive appraisal processes remains underexplored in the existing literature.

Our findings reveal that the effects of psychological resilience vary across different cognitive pathways. Specifically, the positive relationship between algorithmic management and challenge appraisal was stronger at higher levels of psychological resilience. The corresponding positive indirect relationship between algorithmic management and workplace wellbeing through challenge appraisal was also stronger at higher levels of psychological resilience. This suggests that gig workers with higher psychological resilience may be more likely to perceive algorithmic management as a source of valuable resources and opportunities. They may interpret algorithmic feedback and task guidance as mechanisms that support skill development and goal attainment.

However, unlike the positive pathway discussed above, H7c and H7d were not supported. Specifically, psychological resilience did not significantly moderate the relationship between algorithmic management and threat appraisal. It also did not significantly moderate the indirect relationship between algorithmic management and workplace wellbeing through threat appraisal. Although this negative indirect relationship was significant at different levels of psychological resilience, its strength did not vary significantly across these levels. These findings reveal an asymmetric role of psychological resilience across different cognitive appraisal pathways. It should be noted that moderation effects are generally more difficult to detect than main effects, particularly when the magnitude of interaction effects is relatively small. Therefore, the non-significant results of H7c and H7d may partly reflect the limitations of statistical power to detect small interaction effects.

Nevertheless, we argue that this finding does not mean that psychological resilience is ineffective in algorithmic work environments. Instead, it suggests that psychological resilience may not operate uniformly across different cognitive appraisal processes. Psychological resilience can help workers develop more positive challenge appraisals, but it may not fully attenuate threat perceptions arising from algorithmic management. This finding further reveals that the effects of psychological resilience may depend on the specific cognitive appraisal processes involved.

For food delivery workers, high psychological resilience may help them interpret algorithmic feedback and task guidance more positively and view them as resources for improving work capabilities and achieving work goals. However, threat perceptions arising from the controlling demands of algorithmic management may still be influenced by external work conditions. Therefore, the role of psychological resilience may not function as a universal buffer against all stress responses.

Third, this study extends research on the boundary conditions of psychological resilience by revealing the limitations of its effects in algorithmic work environments. Prior research has shown that the positive effects of psychological resilience may depend on the type and duration of stressors, as well as the specific situational context (Mahdiani and Ungar, 2021; Chamorro-Premuzic and Lusk, 2017; Scott et al., 2018). For example, Mahdiani and Ungar (2021) and Chamorro-Premuzic and Lusk (2017) have argued that higher levels of resilience may alleviate stress in the short-term, but may not protect them from the negative consequences of long-term stress. Scott et al. (2018) also found that physicians who already exhibit high levels of resilience still experienced burnout and secondary traumatic stress. These studies further suggest that the benefits of psychological resilience may depend on the duration of stress and the specific context.

This study further extends the boundary conditions of psychological resilience to the context of algorithmic work. Unlike traditional workplace stressors, algorithmic management possesses both resource-enhancing and control-oriented attributes. On the one hand, the informational support, task guidance, and performance feedback provided by algorithmic systems can help workers understand work requirements, improve their work capabilities, and facilitate the formation of challenge appraisals. On the other hand, algorithmic monitoring, performance requirements, and reward and penalty mechanisms may directly influence gig workers' task allocation, income opportunities, and work autonomy (Wood et al., 2019). Therefore, in terms of threat appraisal, workers face not only subjective experiences of stress but also external constraints associated with actual working conditions. This helps explain why psychological resilience can facilitate more positive challenge appraisals among workers but may not fully attenuate threat perceptions arising from algorithmic control mechanisms.

Furthermore, the effectiveness of individual psychological resources may also depend on broader working conditions and structural factors. Past studies have shown that about 90% of food delivery workers in China come from rural areas (Huang, 2022; Lei, 2021). Some of these workers also face social inequalities (such as inadequate social security and household registration restrictions), as well as unstable working conditions imposed by platforms (such as income volatility and algorithmic labor control). Under these conditions, individual psychological resources alone may not be sufficient to offset persistent external constraints. This finding echoes the critique by Masten and Wright (2010) that resilience should be understood in relation to individuals' social and environmental conditions.

It should be noted that the present study did not directly measure the specific processes related to persistent external control or structural constraints. Therefore, these explanations remain theoretical and should be further examined in future research.As numerous researchers in the resilience literature have noted, psychological resilience remains a concept that is not yet fully understood (Ungar, 2012; Rutter, 2012). Therefore, future research needs to further explore the boundaries of psychological resilience's role in different institutional environments, labor relations, and management regimes. Particular attention should be paid to when individual psychological resources can provide protection and when their effects may be constrained by external conditions in highly unstable, low-security, and highly controlled work contexts. Such efforts can provide a more comprehensive understanding of the complex role of psychological resilience in the gig economy.

### 5.2 Practical implications

First, based on our findings, we suggest that food delivery platforms should pay attention to how gig workers cognitively appraise algorithmic management when designing and implementing such systems, rather than focusing only on algorithmic efficiency. The post-hoc analysis shows that support-oriented functions, such as assessment and feedback and allocation and pricing, are more closely associated with challenge appraisal, whereas control-oriented functions, such as tracking and monitoring, are more closely associated with threat appraisal. Therefore, platforms should adopt differentiated approaches when developing and refining different algorithmic functions. For support-oriented functions, platforms can strengthen their enabling role by improving the quality and timeliness of performance feedback, providing clearer task guidance, and ensuring fairer allocation and pricing mechanisms. These practices may help workers better understand algorithmic requirements and perceive algorithm-related demands as manageable work challenges. For control-oriented functions, platforms should place greater emphasis on transparency and procedural fairness. Specifically, platforms can enhance the transparency of tracking and monitoring rules, provide channels for workers to seek explanations and appeal algorithmic decisions, and avoid excessive reliance on punitive management mechanisms. These practices may help reduce threat perceptions associated with algorithmic control.

Second, our findings suggest that platforms should not view psychological resilience as a personal resource that can fully compensate for the pressures associated with algorithmic management. Although psychologically resilient gig workers may be more likely to interpret algorithmic management as a source of resources and opportunities, our findings indicate that resilience alone may not be sufficient to significantly reduce threat perceptions arising from algorithmic management. Therefore, platforms should not rely solely on enhancing gig workers' individual coping abilities but should also consider improving the design of algorithmic management systems themselves. For example, platforms can regularly evaluate how algorithmic monitoring, performance requirements, and reward-penalty mechanisms shape gig workers' experiences under algorithmic management, and incorporate greater flexibility and feedback channels into algorithmic systems. These practices may help reduce potential sources of pressure associated with algorithmic management and promote more sustainable platform work arrangements.

## 6 Limitations and further research

We acknowledge that our study has several limitations, which may also provide new directions for future research. First, the research sample of this study was entirely composed of food delivery workers. However, workers on different online platforms (e.g., Uber, Helpling) may have different focuses when evaluating algorithmic management, which may lead algorithmic management to be perceived more positively or negatively in different contexts. Future research should conduct in-depth investigations on workers in different industries, platforms, and types, so as to expand the conclusions of this study and enhance the generalizability of the findings.

Second, although data were collected at two different time points, and multiple *post hoc* tests did not identify serious common method bias, the two-wave research design cannot fully overcome the limitations of causal inference. This limitation is particularly important for the present study. Based on stress appraisal theory, we proposed a specific mechanism in which algorithmic management acts as a stressor, shapes gig workers' cognitive appraisals, and further influences their workplace wellbeing. However, because this study adopted an observational research design and some variables were measured through self-reports, alternative explanations cannot be completely ruled out. For example, workers with lower workplace wellbeing may develop more negative perceptions of algorithmic management. Therefore, although the temporal separation of variables provides stronger evidence than a single-wave cross-sectional design, the causal relationships among algorithmic management, cognitive appraisals, and workplace wellbeing should still be interpreted with caution. Future research could adopt longitudinal designs, experience sampling methods, or experimental approaches to provide stronger evidence for causal relationships.

Third, prior research suggests that individuals' workplace wellbeing may shape their cognitive appraisals of the work environment, which subsequently influence their work states. However, this reciprocal process has not been widely validated through empirical research. Accordingly, adopting a dynamic perspective on workplace wellbeing is both necessary and timely. Future studies on gig workers' workplace wellbeing could integrate self-reported and other-reported measures, complemented by qualitative evidence, to examine whether and how temporal fluctuations in workplace wellbeing are associated with other work-related outcomes over time.

Fourth, this study focuses on the Chinese context, which may limit the generalizability of the findings. A lot of literature has shown that gig workers in China, particularly food delivery workers, predominantly come from rural areas. Due to structural constraints specific to the Chinese institutional environment, such as the household registration (hukou) system, even workers with high levels of psychological resilience often have limited capacity to avoid or resist unfair treatment from both societal and platform-level actors. Under such conditions, the functioning of psychological resilience may differ from that observed in other institutional settings. Given the cross-national differences in social security systems, labor regulations, and platform governance regimes, future research could adopt comparative designs to examine the contextual applicability and broader validity of the present findings across different economic and cultural contexts.

Fifth, sample attrition represents another important limitation of this study. The unmatched cases from the first wave of data collection were not retained during the data matching and cleaning process. Therefore, we were unable to further examine whether there were systematic differences between retained and attrited participants in terms of demographic characteristics, perceived algorithmic management, psychological resilience, and other study variables. This limitation is particularly important because this study further examined the moderating role of psychological resilience across different cognitive appraisal pathways. Potential attrition bias may have influenced the estimated relationships among algorithmic management, challenge appraisal, threat appraisal, and workplace wellbeing, particularly the interpretation of the moderating effects of psychological resilience. Although the differences in sample retention rates across the three cities were not statistically significant, the possibility of systematic sample attrition cannot be completely ruled out. Therefore, we carefully considered this limitation when interpreting the findings and avoided making causal claims beyond what the research design could support. Future research could adopt more effective sample retention strategies while protecting participants' privacy and collect relevant information from participants who do not complete follow-up surveys to further examine potential attrition bias. In addition, this study did not collect identifying information at the delivery station or platform level during data collection. Therefore, we were unable to examine potential clustering effects at the station or platform level. This limitation may affect the estimation of standard errors and statistical inferences. Future research could retain necessary group-level information while ensuring participants' privacy and apply multilevel modeling or cluster-robust standard error approaches to further improve the robustness of research findings.

## Data Availability

The raw data supporting the conclusions of this article will be made available by the authors, without undue reservation.
