# Supplementary material for: Algorithmic management and gig workers' workplace wellbeing: the moderating role of psychological resilience
Source: Front Psychol. 2026 Oct 15;17:1960382. doi: 10.3389/fpsyg.2026.1960382 (PMC13716670; doi:10.3389/fpsyg.2026.1960382)
Supplement: Supplementary file 1 [file Supplementary_File_1.docx]

**Appendix A**

*Appendix A.1*

**Table A1. Measurements of all variables.**

| Constructs | ID | Measurement | Sources |
| --- | --- | --- | --- |
| Algorithmic Management (Assessment and Feedback) | AF1 | The platform system will comprehensively evaluate my work performance based on customer feedback | Jiang et al., (2025) |
|  | AF2 | The platform system will give me real-time feedback on my work completion |  |
|  | AF3 | When there is an incentive campaign on the platform, the algorithm evaluates my job performance and calculates the level of monetary rewards |  |
|  | AF4 | The platform system evaluates my reputation level based on my job performance. (e.g., level upgrades, etc.) |  |
| Algorithmic Management (Training and Guidance) | TG1 | The platform system provides me with real-time  guidance related to work tasks, such as continuous route navigation and time estimation. | Jiang et al., (2025) |
|  | TG2 | The platform system provides me with online training on work safety and workflow and evaluates the effectiveness of the training (e.g., exams, etc.) |  |
|  | TG3 | The platform system provides me with online training on customer communication skills and conducts training effectiveness assessment (e.g., exams, etc.) |  |
|  | TG4 | In the course of completing a task, the platform  system notifies me when customer needs change and provides guidance on the task |  |
| Algorithmic Management (Allocation and Pricing) | AP1 | The platform system intelligently assigns work tasks to me based on peak task times | Jiang et al., (2025) |
|  | AP2 | The platform system automatically calculates task pricing based on task distance and weather conditions |  |
|  | AP3 | The platform system will assign work tasks based on the completion of my past tasks |  |
| Algorithmic Management (Tracking and Monitoring) | TM1 | The platform system will record in real time information on my behaviour and attitude in completing my work | Jiang et al., (2025) |
|  | TM2 | The platform system will from time to time carry  out random inspection and testing of my dress code, safety equipment and its work norms |  |
|  | TM3 | During work, the platform system will issue alerts for behaviors that do not align with work requirements |  |
| Challenge Appraisal | CA1 | Algorithmic management helps me overcome difficulties at work. | LePine et al., (2016) |
|  | CA2 | Algorithmic management helps me achieve my personal work goals. |  |
|  | CA3 | Overall, algorithmic management has enhanced my personal achievements. |  |
| Threat Appraisal | TA1 | Algorithmic management limits my ability to achieve personal goals and development. | LePine et al., (2016) |
|  | TA2 | Algorithmic management hinders my personal growth and well-being. |  |
|  | TA3 | Overall, algorithmic management threatens my personal achievements. |  |
| Workplace Well-being | WWB1 | I am satisfied with my work responsibilities. | Zheng et al., (2015) |
|  | WWB2 | In general, I feel fairly satisfied with my present job. |  |
|  | WWB3 | I find real enjoyment in my work. |  |
|  | WWB4 | I can always find ways to enrich my work. |  |
|  | WWB5 | Work is a meaningful experience for me. |  |
|  | WWB6 | I feel basically satisfied with my work achievements in my |  |
| Psychological Resilience | PR1 | When facing difficulties at work, I can always find a way to solve them. | Luthans & Youssef, (2007) |
|  | PR2 | When I encounter setbacks at work, I find it hard to recover quickly. |  |
|  | PR3 | I am usually able to handle work pressure calmly. |  |
|  | PR4 | I feel that I am unable to cope with work pressure. |  |
|  | PR5 | When things go wrong at work, I find it difficult to let the challenges completely knock me down. |  |
|  | PR6 | When problems arise at work, I always try to address them proactively. |  |

**Appendix B**

*Appendix B.1 Robustness Analysis with Control Variables*

*Table B1–B3*

**Table B1 Fitting result indicators of theoretical model**

| Common indicators | χ2 | *df* | χ2/*df* | GFI | RMSEA | NFI | CFI | TLI | IFI |
| --- | --- | --- | --- | --- | --- | --- | --- | --- | --- |
| Criteria for judgment | - | - | <3 | >0.9 | <0.10 | >0.9 | >0.9 | >0.9 | >0.9 |
| Value | 950.528 | 456 | 2.084 | 0.876 | 0.053 | 0.827 | 0.901 | 0.892 | 0.902 |

**Table B2 Hypothesis testing results**

| Variable relationship | Path coefficient | S.E. | C.R. | P | Test results |
| --- | --- | --- | --- | --- | --- |
| Algorithmic management → Challenge Appraisal | 0.499 | 0.108 | 7.231 | *** | Supported |
| Algorithmic management → Threat Appraisal | 0.324 | 0.103 | 5.066 | *** | Supported |
| Challenge appraisal → Workplace Well-being | 0.454 | 0.048 | 7.456 | *** | Supported |
| Threat appraisal → Workplace Well-being | -0.208 | 0.042 | -3.816 | *** | Supported |

Note: *p＜0.050,**p＜0.010,***p＜0.001.

**Table B3: Results of mediation effect test**

| Impact Path | Effect | Boot SE | 95%Boot | | P |
| --- | --- | --- | --- | --- | --- |
|  |  |  | LLCI | ULCI |  |
| Algorithmic management → Challenge Appraisal → Workplace Well-being | 0.227 | 0.043 | 0.153 | 0.328 | *** |
| Algorithmic management → Threat Appraisal → Workplace Well-being | -0.067 | 0.022 | -0.118 | -0.032 | *** |

Note: *p＜0.050,**p＜0.010,***p＜0.001.

*Appendix B.2 Alternative Partial Mediation Model*

*Table B4–B6*

**Table B4: Fitting result indicators of theoretical model**

| Common indicators | χ2 | *df* | χ2/*df* | GFI | RMSEA | NFI | CFI | TLI | IFI |
| --- | --- | --- | --- | --- | --- | --- | --- | --- | --- |
| Criteria for judgment | - | - | <3 | >0.9 | <0.10 | >0.9 | >0.9 | >0.9 | >0.9 |
| Value | 943.585 | 455 | 2.074 | 0.877 | 0.053 | 0.828 | 0.902 | 0.893 | 0.903 |

**Table B5: Hypothesis testing results**

| Variable relationship | Path coefficient | S.E. | C.R. | P | Test results |
| --- | --- | --- | --- | --- | --- |
| Algorithmic management → Challenge Appraisal | 0.487 | 0.107 | 7.085 | *** | Supported |
| Algorithmic management → Threat Appraisal | 0.329 | 0.103 | 5.130 | *** | Supported |
| Challenge appraisal → Workplace Well-being | 0.355 | 0.053 | 5.240 | *** | Supported |
| Threat appraisal → Workplace Well-being | -0.265 | 0.045 | -4.528 | *** | Supported |
| Algorithmic management → Workplace Well-bing | 0.191 | 0.088 | 2.648 | ** | Supported |

Note: *p＜0.050,**p＜0.010,***p＜0.001.

**TableB6 : Results of mediation effect test**

| Impact Path | Effect | Boot SE | 95%Boot | | P |
| --- | --- | --- | --- | --- | --- |
|  |  |  | LLCI | ULCI |  |
| Algorithmic management → Workplace Well-being (total effct) | 0.277 | 0.069 | 0.137 | 0.408 | *** |
| Algorithmic management → Workplace Well-being (direct effect) | 0.191 | 0.073 | 0.043 | 0.328 | * |
| Algorithmic management → Challenge Appraisal → Workplace Well-being | 0.173 | 0.038 | 0.111 | 0.271 | *** |
| Algorithmic management → Threat Appraisal → Workplace Well-being | -0.087 | 0.026 | -0.149 | -0.045 | *** |

Note: *p＜0.050,**p＜0.010,***p＜0.001.
